# Evaluating Sample-Based Krylov Quantum Diagonalization for Heisenberg Models with Applications to Materials Science

**DOI:** 10.3390/e28040367

**Published:** 2026-03-24

**Authors:** Neel Misciasci, Roman Firt, Jonathan E. Mueller, Triet Friedhoff, Chinonso Onah, Aaron Schulze, Sarah Mostame

**Affiliations:** 1 Volkswagen AG, Berliner Ring 2, 38440 Wolfsburg, Germanyaaron.schulze@volkswagen.de (A.S.); 2 CIT School, Technical University of Munich, 80333 Munich, Germany; 3 IBM Quantum, IBM Chicago Office, Chicago, IL 60606, USA; triet.nguyen-beck@ibm.com; 4 Department of Physics, RWTH Aachen University, 52062 Aachen, Germany; 5 Department of Physics, University of Ulm, Albert–Einstein–Allee 11, 89081 Ulm, Germany; 6 IBM Quantum, IBM T.J. Watson Research Center, Yorktown Heights, NY 10598, USA

**Keywords:** quantum computing, sample-based quantum diagonalization methods, spin models, quantum-centric supercomputing architectures

## Abstract

We evaluate the Sample-based Krylov Quantum Diagonalization (SKQD) algorithm on one- and two-dimensional Heisenberg models, including strongly correlated regimes in which the ground state is dense. Using problem-informed initial states and magnetization sector sweeps, we investigate SKQD for problems with non-sparse ground states, where energy accuracy and sampling efficiency are theoretically anticipated to degrade. Our studies reveal that SKQD reproduces ground-state energies and field-dependent magnetization across a range of anisotropies. Benchmarks against DMRG and exact diagonalization show consistent qualitative agreement, with accuracy improving systematically in more anisotropic regimes. We further demonstrate SKQD on quantum hardware by implementing 18- and 30-qubit Heisenberg chains, obtaining magnetization curves that match theoretical expectations. Simulations on the IBM Nighthawk processor for 64-qubit two-dimensional square lattice systems further indicate that the method remains effective beyond one-dimensional geometries.

## 1. Introduction and Background

The Heisenberg model is a cornerstone of quantum magnetism, capturing the essential physics of interacting localized spins. It serves as a fundamental framework for understanding quantum many-body phenomena such as entanglement, quantum phase transitions, and emergent collective behavior in both low- and higher-dimensional systems. In particular, the spin-$\frac{1}{2}$ Heisenberg Hamiltonian provides a minimal yet non-trivial setting for studying strongly correlated quantum matter that can be probed analytically, simulated numerically, and realized experimentally on both classical and quantum platforms. Beyond its historical importance, the quantum Heisenberg model has become a central benchmark for the development of quantum simulation protocols on quantum hardware [1,2,3,4,5]. Its tunable ground-state structure, ranging from critical entangled states in the gapless Luttinger liquid regime to trivially polarized states under strong magnetic fields, offers a unique testbed for quantum algorithms. Variations in anisotropy and external fields control the transition between sparse and non-sparse representations of the Hamiltonian, making the model a suitable test-case for evaluating algorithmic robustness and scalability on quantum devices.

The generic Heisenberg Hamiltonian for a lattice of spin-$\frac{1}{2}$ particles is given by

(1) $$H=J\;\sum_{\langle i,j\rangle }\left(S_{i}^{x}S_{j}^{x}+S_{i}^{y}S_{j}^{y}+\Delta \;S_{i}^{z}S_{j}^{z}\right)-\sum_{i}\vec{h}\cdot {\vec{S}}_{i}$$

where $S^{\alpha }\left(\alpha =x,y,z\right)$ are Pauli matrices, 〈i,j〉 denotes pairs of neighboring spins, $\vec{h}$ is an external magnetic field, with *J* being the exchange coupling constant; J>0 represents the antiferromagnetic (AFM) coupling, while J<0 describes the ferromagnetic (FM) coupling. Δ is the anisotropy parameter; Δ=1 describes the isotropic Heisenberg model (XXX model) while Δ≠1 represents the anisotropic model (XXZ model). Values of the exchange coupling *J* and the anisotropy parameter Δ found in real spin-$\frac{1}{2}$ quasi-1D quantum magnets are listed in Table 1. Values reaching from easy-plane XX-anisotropy with Δ<1 to isotropic materials with Δ=1 up to Ising-type anisotropy with Δ≫1, which shows that the full range of anisotropies in the XXZ model is significant for real materials.

A magnetic field applied along the *z*-axis adds a Zeeman term $H_{z}=-h_{z}\sum_{i}S_{i}^{z}\;,$ which preserves the U(1) symmetry associated with $S_{tot}^{z}$, but breaks the SU(2) symmetry in the isotropic case. It shifts the energy of spin-up vs. spin-down states and can polarize the system. In the strong-field limit, the ground state becomes fully polarized, leading to a trivial product state. At weaker fields, the ground state remains highly entangled, but becomes partially polarized. In the 1D Heisenberg model in Zeeman external field, the system exhibits a quantum phase transition as magnetization increases with field strength. A magnetic field applied perpendicular to the quantization axis (e.g., in the *x*-direction) introduces a transverse term $H_{\mathrm{tr}}=-h_{x}\sum_{i}S_{i}^{x}\;.$ This term does not commute with $S_{z}$, leading to quantum fluctuations that can destroy magnetic order. In the anisotropic XXZ and Ising limits, transverse fields can induce quantum phase transitions, such as from a Néel-ordered phase to a paramagnet. The transverse-field Ising model is a particularly well-studied limiting case with rich critical behavior in 1D and 2D. The magnetization of a quantum spin system quantifies the degree of alignment of spins with respect to a given direction, and it serves as a key observable in characterizing magnetic-order and phase transitions.

Despite extensive classical studies of quantum spin systems, the numerical simulation of strongly correlated Hamiltonians with non-sparse ground states remains computationally demanding beyond one dimension. This challenge is closely tied to the scaling of the entanglement entropy in many-body states. For ground states of gapped local 1D Hamiltonians, the bond dimension required for fixed accuracy in Density Matrix Renormalization Group (DMRG) simulations is independent of the system size; however, in 2D, it scales exponentially with the system width [12]. Although Projected Entangled Pair States (PEPSs) naturally accommodate 2D area laws, their contraction is #P-hard, rendering the simulation of large general systems intractable [13]. These classical limitations highlight the need for alternative approaches, such as those offered by quantum computing.

In this work, we extend this line of research by evaluating the recently developed *Sample-based Krylov Quantum Diagonalization* (SKQD) [14] algorithm on the Heisenberg model. The SKQD approach belongs to the emerging class of sample-based quantum diagonalization methods, designed for efficient implementation on quantum-centric supercomputing architectures [15,16,17]. Although the Heisenberg model does not inherently satisfy the sparsity requirement assumed in the formal efficiency analysis of SKQD, we develop a systematic strategy for adapting the algorithm to strongly correlated Hamiltonians with a dense ground-state structure. Remarkably, this enables reliable estimation of observables such as magnetization across multiple regimes of the phase diagram, including both isotropic and anisotropic interactions and in the presence of external magnetic fields. Our results suggest that SKQD can be applied beyond the sparsity assumptions underlying its theoretical efficiency analysis, without an immediate loss of practical performance. This work thus connects algorithmic innovation with physically rich test systems, bridging current quantum computational techniques and foundational models of quantum magnetism.

## 2. Method

Our study focuses on the Sample-based Krylov Quantum Diagonalization (SKQD) algorithm [14], a recent advance in hybrid quantum–classical computation for the spectral estimation of quantum many-body systems. SKQD determines the low-energy spectrum by constructing and diagonalizing an effective Hamiltonian in a Krylov subspace, where all matrix elements are obtained directly from sampled quantum measurements rather than explicit state vectors. This strategy enables scalable and noise-resilient spectral estimation that circumvents the exponential overhead associated with full-state tomography. Developed to mitigate the limitations of current quantum hardware, SKQD unifies key elements from two complementary approaches: Krylov Quantum Diagonalization (KQD) [4] and Sample-based Quantum Diagonalization methods (SQD) [18,19,20,21]. KQD constructs a Krylov subspace via short-time unitary evolution followed by classical diagonalization, while SQD approximates ground-state energies through direct sampling and classical post-processing for error mitigation. SKQD integrates these concepts, inheriting the convergence guarantees and noise-tolerant characteristics of both. As a result, it is particularly well suited for near-term quantum devices and for strongly correlated problems such as quantum magnetism and material simulations.

**Figure 1:**
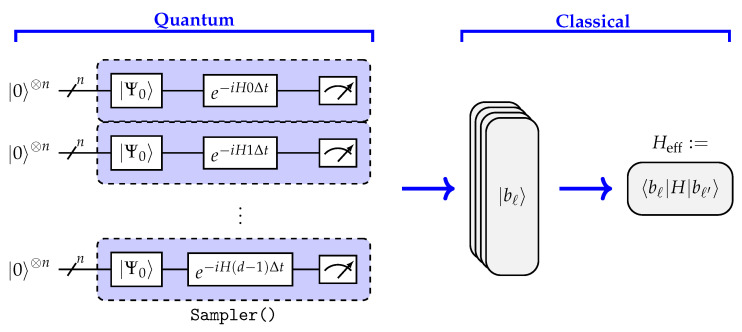
Construction of projected Hamiltonian $H_{\mathrm{eff}}$ from bitstrings $|b_{\ell }\rangle$ after being sampled from Krylov evolution quantum circuits.

In SKQD, the effective Hamiltonian is constructed within the quantum Krylov subspace generated by time-evolved reference states. For a system described by a Hamiltonian *H* acting on an $N=2^{n}$-dimensional Hilbert space of *n* qubits and an initial state $|\psi_{0}\rangle$, the Krylov basis is defined as

(2) $$|\psi_{k}\rangle =e^{-ikH\Delta t}|\psi_{0}\rangle ,\;k=0,1,\ldots ,d-1,$$

where Δt is a discrete time step and *d* denotes the chosen subspace dimension [14]. Instead of estimating Hamiltonian matrix elements $\langle \psi_{i}|H|\psi_{j}\rangle$ through controlled measurements, SKQD samples bitstrings $|b_{\ell }\rangle$ directly from the Krylov states on the quantum device. The collected samples define a reduced configuration space, in which the Hamiltonian is projected, ${\left(H_{\mathrm{eff}}\right)}_{\ell \ell^{\prime }}=\langle b_{\ell }\left|H\right|b_{\ell^{\prime }}\rangle$ and subsequently diagonalized using a classical computer, obtaining approximations of the low-energy eigenvalues and eigenstates [14]. Figure 1 illustrates the workflow of the SKQD approach.

This strategy combines the systematic convergence properties of Krylov quantum diagonalization with the robustness and shallow-circuit requirements of sample-based methods. The efficiency of this basis construction and the convergence of the estimated energy depend on structural properties of the Hamiltonian and its ground state. The SKQD performance guarantees require the Hamiltonian, its ground state, and the Krylov evolution initial state to obey certain requirements, such as a well-behaved spectral property, non-trivial initial state overlap with the true ground state and ground-state sparsity. Let us recall the sparsity definition from [14]:

**Definition 1**  (($\alpha_{L}$, $\beta_{L}$)-sparsity). *For any state *|*ψ*〉 *let*

(3) $$|\psi \rangle =\sum_{k}g_{k}|b_{k}\rangle$$

*with ordering *$|g_{1}\left|\;\geq \;\right|g_{2}|\;\geq \ldots$. *We say that* |*ψ*〉 *exhibits* $\left(\alpha_{L},\beta_{L}\right)$-*sparsity if*

(4) $$\sum_{k=1}^{L}{\left|g_{k}\right|}^{2}\geq \alpha_{L}$$

*and*

(5) $$|g_{k}{|}^{2}\geq \beta_{L},\;1\leq k\leq L.$$

**Figure 2:**
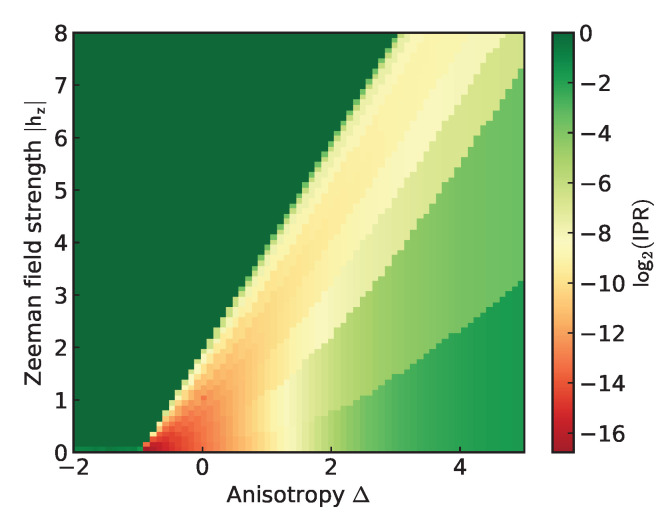
Illustration of ground-state sparsity for 20-spin system with J=1 in $\left(\Delta ,h_{z}\right)$-space plotted as the logarithm of the inverse participation ratio.

The sparsity parameters $\alpha_{L}$ and $\beta_{L}$ prove that the ground-state energy can be approximated in polynomial time, under assumptions of Krylov quantum diagonalization and sparseness of the ground state [14]. We refer to Appendix A for a more detailed overview. To further understand these requirements in practice, it is useful to examine how the underlying state representation behaves under different physical regimes. To illustrate how the sparsity of the ground state of (1) changes with respect to the anisotropy and strength of the external magnetic field, Figure 2 plots the logarithm of the Inverse Participation Ratio (IPR) of the ground state in the $\left(\Delta ,h_{z}\right)$ space for a 20-spin system.

For an *N*-qubit quantum state $|\Psi \rangle =\sum_{k=0}^{2^{N}-1}g_{k}|k\rangle$, IPR is defined as

(6) $$\mathrm{IPR}\left(|\Psi \rangle \right):=\sum_{k=0}^{2^{N}-1}{\left|g_{k}\right|}^{4}$$

and is equal to 1 for |Ψ〉 concentrated in a single bitstring and to $2^{-N}$ for a uniform superposition of all possible bitstrings. Hence, it can serve as an indicator of the ground state’s basis bitstring concentration.

Figure 2 could be interpreted as a discrete version of the familiar XXZ-Heisenberg Hamiltonian phase diagram that clearly illustrates the trivial ferromagnetic region and the transition from a gapped system to a gapless critical region depending on the particular choice of $\left(\Delta ,h_{z}\right)$. The discontinuities in the figure mark individual particle/magnetization sectors of the 20 qubit systems. The color spectrum of Figure 2 thus naturally specifies the regimes where SKQD performance might struggle due to the lack of ground state sparsity.

Compared with standard variational quantum eigensolver (VQE) techniques, SKQD eliminates the need for parameter optimization, avoiding vulnerability to barren-plateau effects and lowers sensitivity to initialization. Instead, its performance depends primarily on controlled time-evolution accuracy and sampling precision, both of which scale favorably on near-term quantum hardware. This combination of algorithmic simplicity, robustness to noise, and spectral accuracy positions SKQD as a promising framework for quantum simulations of correlated spin systems and Hamiltonians with dense ground-state structure.

## 3. Benchmark Setup

### 3.1. Model Hamiltonians and Lattice Geometries

We benchmark the SKQD algorithm on the spin-$\frac{1}{2}$ Heisenberg family in both one and two spatial dimensions. Open boundary conditions are used throughout. In 1D every qubit represents one lattice site of a linear chain. For 2D lattices, an *N*-qubit register is mapped onto the *most-square* open rectangle r×c (N=rc with r≤c) so that all qubits have nearest-neighbor connectivity along both axes. The rectangle is chosen by the simple divisor-search heuristic

(7) $$\left(r,c\right)=\underset{\underset{r\leq c}{rc=N}}{\mathrm{argmin}}\left|\;r-c\;\right|,\;r=\max\left\{\;\varrho \leq \sqrt{N}\;|\;N\;\mathrm{mod}\;\varrho =0\right\},$$

where the search starts at $\left\lfloor \sqrt{N}\right\rfloor$ and steps downward until the first divisor ϱ is found; c=N/ϱ follows directly—For prime *N* the loop stops at ϱ=1, and the layout degenerates to a 1×N strip with nearest-neighbor connectivity.

### 3.2. Initial State Preparation

The error in SKQD is determined by the “sparsity” of the ground state, and only *indirectly* tied to the overlap with the initial state. A poor choice of initial state does not worsen the theoretical best-case error bound, but it can make the quantum measurement cost required to reach that bound prohibitively large. For a theoretical understanding we refer again to Appendix A.

#### 3.2.1. Singlet State

The single product state provides a physically-motivated, high-quality first guess, that is known to be adiabatically connected to the true ground state. To introduce the construction more clearly, we begin with the formula and the corresponding circuit for a two-qubit singlet system

$$|\Psi^{-}\rangle =\frac{1}{\sqrt{2}}(|01\rangle -\left|10\rangle \right)=U_{\mathrm{singlet}}|00\rangle$$

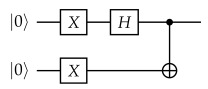




In [1], the authors identify the singlet state as a suitable starting-point ansatz for Δ in the AFM range, arguing that the AFM Heisenberg model favors anti-aligned spins, and a singlet pair $\left(|01\rangle -|10\rangle \right)/\sqrt{2}$ is the quintessential quantum state of two perfectly anti-aligned spins. Although we do not make use of their adiabatic connection, we adopt the same physical reasoning to justify its use in our SKQD simulations.

For many of our numerical experiments, we choose the singlet product state as the initial state

$$|\psi_{\mathrm{singlets}}\rangle =\frac{1}{\sqrt{2^{N/2}}}\prod_{j=1}^{N/2}{\left(|01\rangle -|10\rangle \right)}_{2j-1,2j}\;.$$

This state is the ground state of the Hamiltonian with interaction only on odd bonds, for even N

$${\hat{H}}_{\mathrm{odd}}=\sum_{j=1}^{N/2-1}\left(\sigma_{x}^{\left[2j-1\right]}\sigma_{x}^{\left[2j\right]}+\sigma_{y}^{\left[2j-1\right]}\sigma_{y}^{\left[2j\right]}+\Delta \sigma_{z}^{\left[2j-1\right]}\sigma_{z}^{\left[2j\right]}\right),$$

making it a natural and physically well-motivated choice for our problem setting.

#### 3.2.2. Single Particle Sector Sweep

One disadvantage of the singlet initial state presented above and other half-filled states (like Néel state) is that their half-filled Hamming weight is being propagated along the Krylov evolution; hence, all the bitstrings sampled (in a hypothetical noiseless setting) are going to be half-filled as well, while this works in isotropic AFM Hamiltonian setting (J=1, Δ=1), in case we decide to break the symmetry with a Zeeman field, half-filled initial states are not suitable candidates for constructing a ground state approximation. As a consequence, the quality of the approximated ground-state energy using singlets as the initial state will degrade as the system’s ground state gets more polarized (i.e., shifted away from half-filled). We need to be able to sample bitstrings from different particle sectors as well to make sure we obtain a good approximation of the system’s ground state. Taking into account that our Zeeman field will only be pointing in one direction, we need to sweep through all particle sectors from the zero state to the half-filled states.

**Figure 3:**
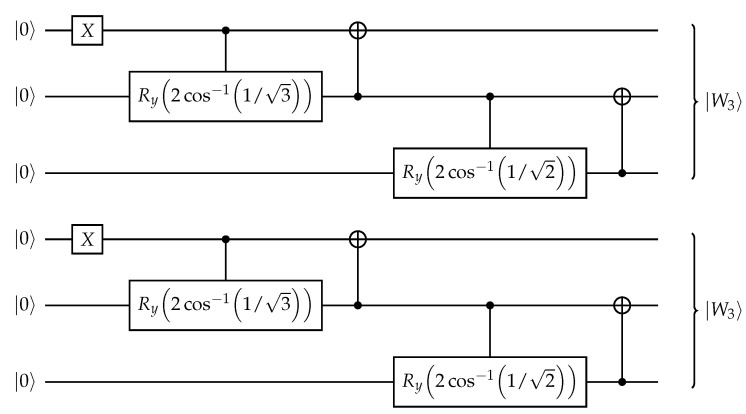
Example initial state (8) for 6 qubits and particle sector k=2, consisting of two 3-qubit W-states. Algorithms 1 and 2 in Ref. [22] realizes this circuit for any given number of blocks and block sizes.

For a particle sector with *k* particles, we divide the qubits into *k* groups and initialize the state as a product of *W*-states, with one in each group (as illustrated in Figure 3):

(8) $$|\psi_{0}^{k}\rangle :=\overset{k-1}{\underset{m=0}{⨂}}|W_{\left\lfloor (m+1)N/k\rceil -\lfloor mN/k\right\rceil }\rangle \;,$$

where ⌊x⌉ is a rounding operation to the nearest integer and $W_{m}$ is an *m* qubit *W*-state defined as

(9) $$|W_{m}\rangle =\frac{1}{\sqrt{m}}\left(|0\ldots 01\rangle +|0\ldots 010\rangle +\ldots +|010\ldots 0\rangle +|10\ldots 0\rangle \right).$$

Being a product of *W*-states the preparation of $|\psi_{0}^{k}\rangle$ can be done efficiently with O(N/k) circuit depth. It is apparent that $|\psi_{0}^{k}\rangle$ contains only *k*-particle bitstrings. Moreover, the choice of *W*-states ensures that the particles are spread evenly as, for example, the |…11…〉 bitstring is not present, favoring the AFM particle ordering.

In order to approximate the ground state magnetization, we first construct an initial state (8) lying in the particle sector of interest. Then we calculate the ground-state energy approximation using SKQD. Since the particle sector is preserved through the evolution, we get (in the noiseless setup) a ground-state energy approximation restricted to a single particle sector. To get the real ground state approximation, we do the sweep for all particle sectors from zero particles (trivial) to half-filled, and whichever has the smallest energy, we designate it to be the ground state approximation of the system. The value of magnetization is then equal to the magnetization characteristic for the particle sector, which delivered the lowest energy in the sweep. To save computational time, we only choose particle sectors close to the value of the reference DMRG calculation for each value of the Zeeman term, since particle sectors far away from the true ground state are unlikely to produce an even lower energy approximation. Please refer to Appendix C for a detailed description.

## 4. Results

The performance of SKQD across the antiferromagnetic and gapless regimes, on one- and two-dimensional Heisenberg models is investigated using a combination of quantum hardware experiments, exact and DMRG benchmarks, and simulations on small 2D lattices. Our results show that SKQD reliably captures ground-state energies and magnetization in the antiferromagnetic phase, where the sparsity is well conditioned. Moreover, even in regimes where the ground state exhibits a dense structure and sparsity is absent, SKQD reproduces the correct qualitative trends, albeit with larger relative error. For the underlying Hamiltonian, we chose the quasi-1D model of Copper Pyrazine Dinitrate (CuPzN) as presented in [7] resulting in isotropic (XXX) Hamiltonian setting with the coupling constant J=0.91meV. The other parameters used to setup and run the SKQD simulations on quantum hardware are reported in Appendix D.

**Figure 4:**
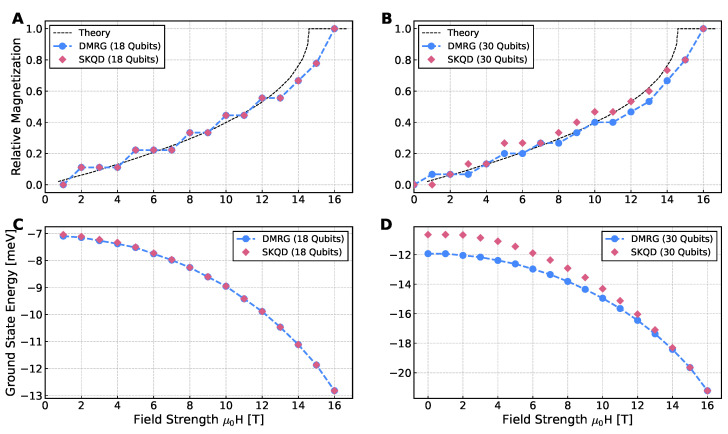
Results from ibm_marrakesh. Relative magnetization (**A**,**B**) and ground-state energy (**C**,**D**) of CuPzN approximated with SKQD for 18 (**A**,**C**) and 30 (**B**,**D**) spin systems. Compared with theoretical values derived from Bethe ansatz [7] and state-of-the art DMRG calculations. We used the initial state described in Section 3.2.2.

Figure 4A,B show the magnetization of CuPzN reconstructed with SKQD on 18- and 30-qubit IBM Heron quantum processor. Across the entire field range, SKQD closely follows the Bethe ansatz reference curve, including the nonlinear rise in the intermediate-field region and saturation near the critical field. This agreement is notable because the low-field isotropic regime corresponds to the hardest region in the sparsity diagram, see Figure 2, where the ground state is highly delocalized.

**Figure 5:**
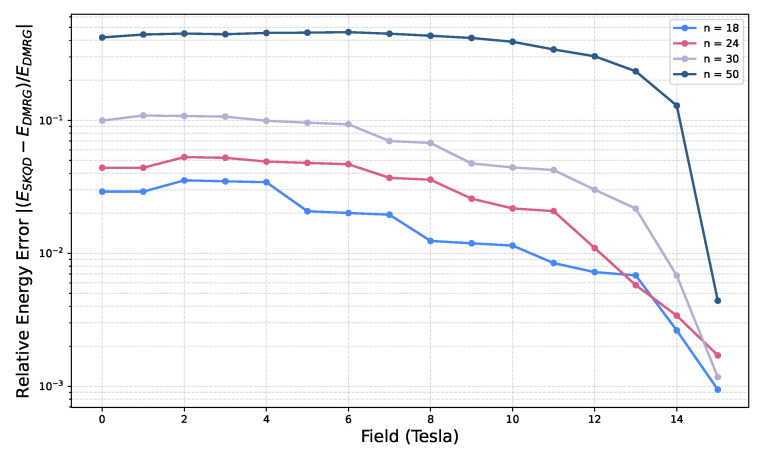
Relative energy error $|\left(E_{\mathrm{SKQD}}-E_{\mathrm{DMRG}}\right)/E_{\mathrm{DMRG}}|$ as a function of magnetic field.

The energy estimates in Figure 4C,D further validate the performance of SKQD. For 18 qubits, SKQD tracks DMRG with excellent accuracy. For 30 qubits, deviations appear in the weak-field regime, consistent with reduced sparsity and increased sampling demands, but systematically diminish as the external field polarizes the ground state. As seen in Figure 2, weaker fields place the system in a challenging regime for SKQD, $\left(\Delta ,h_{z}\right)∼\left(1,0\right)$, which implies increasing classical postprocessing demands as the system size grows. The Inverse Participation Ratio map in Figure 2 provides a unified explanation of the behavior of SKQD. Accuracy is reduced in low-field, isotropic regions where the ground state is highly entangled and non-sparse, but improves distinctly as either field strength or anisotropy increases. To explicitly quantify these boundaries and assess the algorithm’s scalability, we present the relative energy error, $|\left(E_{SKQD}-E_{DMRG}\right)/E_{DMRG}|$, across multiple system sizes in Figure 5. In the highly entangled weak-field regime (<4 T), the sampling overhead becomes a bottleneck, and the error scales noticeably with system size, reaching approximately 30–40% for n=50. Conversely, as the field strength surpasses 10 T and the system shifts toward the sparse, polarized regime, the relative error for all system sizes drops sharply toward a high-accuracy baseline (<$10^{-3}$). This quantitative scaling directly mirrors the theoretical Inverse Participation Ratio transitions previously established in Figure 2. To extract magnetization on hardware, we employ a magnetization sector sweep using W-state initializations tailored to each conserved particle number. Post-selection on excitation number (Appendix B) is critical: although incorrect bitstrings do not affect the minimum-energy estimate, they bias magnetization. Filtering restores correct sector ordering and yields smooth and physically accurate curves.

**Figure 6:**
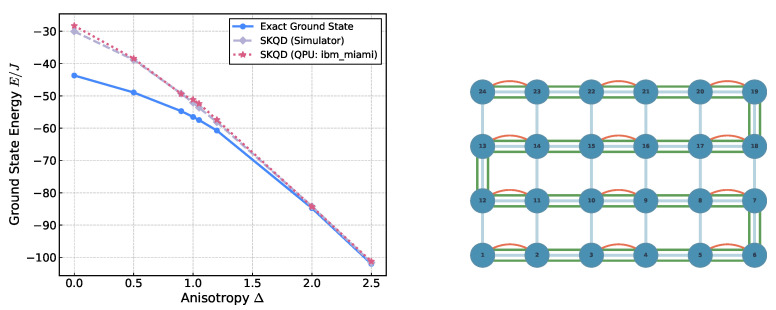
Comparison of SKQD energy estimates from hardware runs on ibm_miami (120-qubit IBM Nighthawk r1), simulations using IBM Aer Simulator, and exact energies for a 6×4 square lattice (**left**). Here we explore the broader phase diagram and algorithmic scaling independent of a specific material; we use dimensionless units by setting the exchange coupling to J=1. The second picture (**right**) represents a snake-like initial state (green) on a 2D square lattice layout used for SKQD simulation. The red lines show the state’s singlet couples.

**Figure 7:**
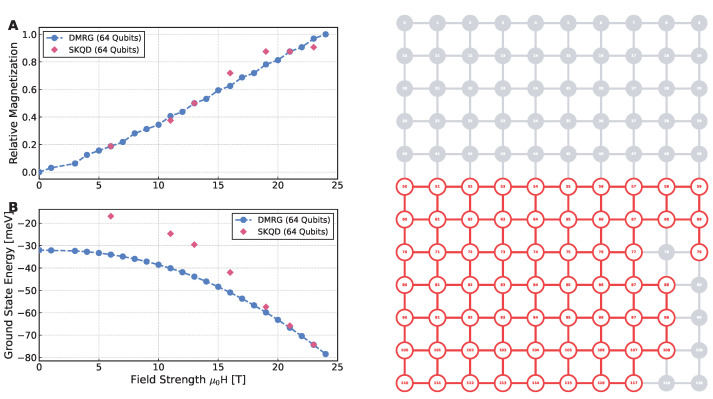
Experimental results and hardware topology. (**left**) Relative magnetization (**A**) and ground-state energy (**B**) of $\left[CuCl{\left(pyz\right)}_{2}\right]\left(BF_{4}\right)$ approximated with SKQD for an 8×8 lattice compared with DMRG [23]. (**right**) Qubit layout and connectivity map of ibm_miami. The red-highlighted region shows an example of the 8×8 lattice employed in our hardware simulations.

Motivated by the availability of the IBM Nighthawk Quantum processor, which features four-nearest-neighbor square lattice connectivity, and to assess performance beyond 1D, we apply SKQD to a 6 × 4 square lattice using a shallow snake-like singlet initialization; see Figure 6. Despite the increased connectivity of the lattice and the dense ground-state structure, SKQD reproduces the overall monotonic dependence of the ground-state energy on anisotropy and captures the correct qualitative trends when compared with exact diagonalization across much of the Δ range. Agreement improves toward the Ising limit, consistent with the sparsity trends observed in one dimension. As a final demonstration, we characterize the magnetization process of an 8×8 spin-$\frac{1}{2}$ lattice by tracking the evolution of the ground-state energy and longitudinal magnetization under an external field. The system is modeled after the $Cu^{2+}$ coordination polymer $\left[CuCl{\left(pyz\right)}_{2}\right]\left(BF_{4}\right)$, a quasi-2D Heisenberg anti-ferromagnet with an isotropic coupling constant J=0.81 meV [23]. Results can be seen in Figure 7. Although hardware noise and the high density of states near the zero-field limit lead to quantitative deviations from DMRG benchmarks, SKQD reproduces the correct qualitative behavior of the magnetization curve. These findings suggest that advanced post-processing techniques could further refine these estimates, potentially reducing the sampling overhead while improving the fidelity of the effective Krylov subspace. Such developments will be critical for scaling SKQD to larger 2D architectures on near-term noisy devices. These results indicate that SKQD can be extended to higher-dimensional lattice geometries while retaining qualitatively reliable performance in the high field regime.

## 5. Conclusions and Outlook

The performance and applicability of the SKQD method on the Heisenberg family of spin-$\frac{1}{2}$ models in both one and two spatial dimensions was evaluated in this work. Despite the strongly correlated nature of these models and the resulting dense ground-state structure, well outside the sparsity assumptions underlying the formal efficiency analysis of SKQD, we find that the method exhibits consistent convergence and captures qualitative trends across regimes ranging from easy-plane anisotropy to the isotropic point and deep into the Ising-like region. This observation highlights the practical applicability of SKQD beyond the sparsity regime assumed in existing analyses. We benchmark SKQD against the Bethe ansatz and DMRG calculations. While numerical discrepancies stemming from finite sampling, approximate Krylov-space construction, and batch truncation naturally remain, the comparison with DMRG demonstrates that SKQD captures the correct physical behavior and that its performance improves systematically as anisotropy increases and effective sparsity becomes more favorable. These benchmarks provide a physically meaningful validation of the algorithm and highlight the regions of the phase diagram where SKQD is most effective and confirm that SKQD remains robust even when sparsity assumptions are not strictly satisfied.

Beyond energy estimation, we explored how initialization strategies influence convergence and observable accuracy. Physically motivated initial states such as singlet products yield strong performance in the antiferromagnetic regime, while W-state sector sweeps enable accurate magnetization extraction by exploiting underlying U(1) symmetry. Our results show that SKQD can reliably identify magnetization sectors and reproduce field-dependent spin polarization trends, providing evidence that the method can access more complex observables than just ground-state energies. Our 2D simulations further illustrate that SKQD extends naturally to higher-dimensional lattice geometries. We demonstrate quantum-hardware results for 18- and 30-qubit 1D Heisenberg chains, as well as for 24- and 64-qubit 2D Heisenberg models. These simulations provide a direct comparison between SKQD on real quantum devices and DMRG baselines, enabling the first hardware demonstration of SKQD applied to magnetization curves and field-driven transitions.

Although the application of SKQD to the antiferromagnetic XXZ Heisenberg Hamiltonian has been illustrated in prior Qiskit tutorial material (https://quantum.cloud.ibm.com/learning/en/courses/quantum-diagonalization-algorithms/skqd) for a 22-qubit system, the present work substantially broadens the methodological and physical scope of the approach. Rather than focusing on a proof-of-principle implementation, we assess SKQD in the context of physically relevant magnetic-material observables, including ground-state energies and field-dependent magnetization curves, with systematic benchmarking against DMRG calculations. We further extend the methodology from one-dimensional chains to two-dimensional square lattices executed on quantum hardware, thereby examining its applicability beyond canonical 1D testbeds. To address simulations in the presence of external fields, we introduce a sector-aware SKQD workflow that enables controlled access to specific magnetization sectors. In addition, we relate algorithmic performance to properties of the ground-state structure, including sparsity characteristics that influence sampling efficiency and convergence. Finally, we report explicit and practically motivated parameter regimes, such as Krylov dimension, time step selection, shot budgets, and hardware configurations, thereby providing reproducible guidance for large-scale implementations beyond tutorial-level default settings, see Appendix D for details.

Overall, our study demonstrates that SKQD is a flexible quantum–classical method for correlated spin systems, capable of scaling for appropriate ground-state sparsity regimes and achieving meaningful agreement with state of the art classical methods such as DMRG while remaining implementable on near-term quantum hardware. With the forthcoming magnetization experiments and extended 2D benchmarks, SKQD stands as a promising tool for quantum simulation in both algorithmic and materials science contexts.

## Figures and Tables

**Table 1:** Reported values of the exchange coupling *J* and anisotropy parameter Δ for selected spin-$\frac{1}{2}$ quasi-1D quantum magnets that can be described with the XXZ-Hamiltonian from Equation (1). To show comparable results, Δ was derived from anisotropy values if other parameters were reported.

Material	J/meV	Δ	Reference
Cs_2_CoCl_4_	0.23	0.25	[6]
CuPzN	0.91	1.00	[7]
KCuF_3_	33.5	1.00	[8]
BaCo_2_V_2_O_8_	3.05	1.90	[9]
SrCo_2_V_2_O_8_	3.7	2.10	[10]
CsCoBr_3_	1.25	6.25	[11]
CsCoCl_3_	0.595	10.42	[11]

**Figure A1:**
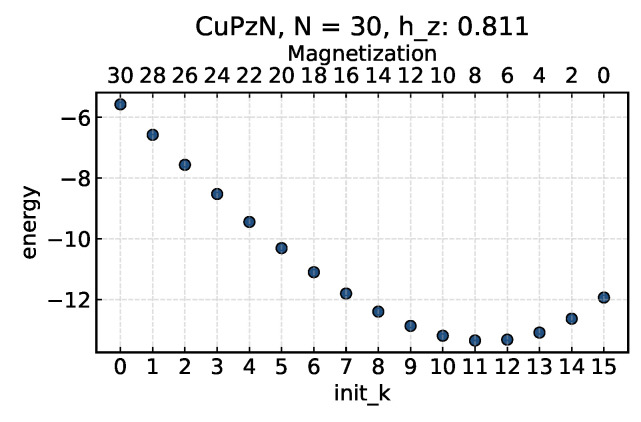
Example of DMRG magnetization sector sweep in the presence of external field.

**Figure A2:**
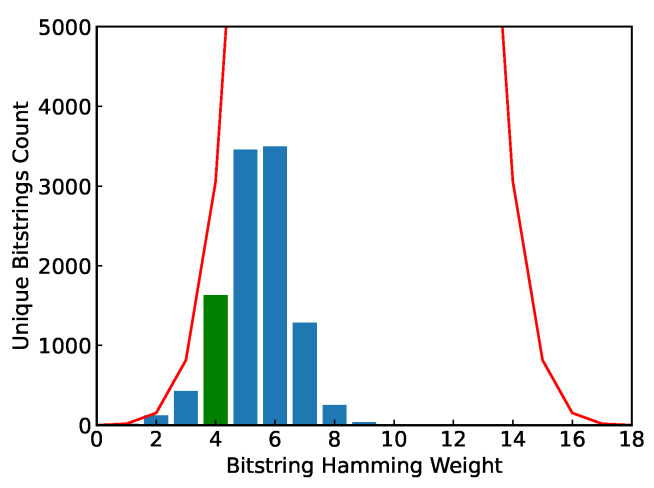
Histogram of sampled unique bitstrings on 18 spin system. Green bar marks the initialization particle sector and red line shows the maximum number of unique bitstrings, explaining the sampling distribution mean value shift towards the half-filling sector in the middle.

## Data Availability

The original contributions presented in this study are included in the article. Further inquiries can be directed to the corresponding authors.

## Appendix A. SKQD Requirements

The following theoretical bounds have been showed in [14]. Let *L* be the smallest possible integer and $\alpha_{L}^{\left(0\right)}$ and $\beta_{L}^{\left(0\right)}$ be the largest possible parameters such that the ground state exhibits $\left(\alpha_{L}^{\left(0\right)},\beta_{L}^{\left(0\right)}\right)$ sparsity. To better understand the resource requirements of the algorithm, we first define three key parameters related to the system’s spectral properties and our initial setup:

- *d* is the dimension of the Krylov subspace, which corresponds to the number of time-evolved states $|\psi_{k}\rangle$ used to generate the subspace.
- $\gamma_{0}$ represents the coefficient of the true ground state $|\phi_{0}\rangle$ in the eigenstate decomposition of the initial reference state $|\psi_{0}\rangle$. Consequently, $|\gamma_{0}{|}^{2}$ quantifies the overlap of the initial state with the true ground state.
- $\tilde{\varepsilon }$ is an error term that provides an upper bound on the *squared* 2-norm distance ($\left|\right|\tilde{\phi }\rangle -|\phi_{0}{\rangle ||}^{2}\leq \tilde{\varepsilon }$) between the exact ground state and the approximate ground state obtained via Krylov diagonalization. It is defined in relation to the spectral gap ($\Delta E_{1}$) and the Krylov energy error (ε), mathematically reflecting how the proximity to the first excited state impacts the state approximation.

The main theorem from [14] states that the error in the ground-state energy $\Delta E_{0}$ is bounded by $\alpha_{L}^{\left(0\right)}$:

(A1) $$\Delta E_{0}\leq \sqrt{8}\;∥H∥\;{\left(1-\sqrt{\alpha_{L}^{\left(0\right)}}\right)}^{1/2}$$

Note that initial state overlap $|\gamma_{0}{|}^{2}$ is not explicitly mentioned in this final error formula, the accuracy is only guaranteed if we successfully sample all the *L* important bitstrings. The practicality of this is governed by the number of samples M required from each Krylov basis state. The theorem also specifies that M is bound by $\beta_{L}^{\left(0\right)}$:

(A2) $$M>\frac{d^{2}\log\left(L/\eta \right)}{|\gamma_{0}{|}^{2}\;\left(\beta_{L}^{\left(0\right)}-2\sqrt{\tilde{\varepsilon }}\right)}$$

where the number of samples required scales inversely with the overlap of the initial state $|\gamma_{0}{|}^{2}$. In this sample bound, η is the failure probability for sampling all necessary bitstrings. This sampling cost equation effectively dictates the algorithm’s practical feasibility. Crucially, the number of samples *M* required scales inversely with both the initial state overlap $|\gamma_{0}{|}^{2}$ and the sparsity parameter $\beta_{L}^{\left(0\right)}$. This means that the computational overhead will shoot up significantly if there is a poor initial overlap with the ground state, or if the ground state exhibits low sparsity (i.e., its wave function is spread across many bitstrings rather than concentrated in a few). By framing the cost in these terms, it becomes clear that selecting a high-quality reference state and operating in a basis where the target state is sparse are critical for keeping the sampling overhead manageable. In our work, we attempt to satisfy only the first requirement (i.e., we define a suitable reference state), but cannot avoid violating the second one (the target state is dense in our basis).

## Appendix B. Exploring Magnetization Sectors with W States

In a similar fashion to the KQD approach, the SKQD algorithm combined with the use of W states as initial states allows us to predict the correct magnetization in the presence of a longitudinal field $h_{z}$ by explicitly exploiting the underlying U(1) symmetry. We perform a magnetization sector sweep, considering each sector *k* of excited particles separately. Each such sector corresponds to a magnetization

M=N−2k,

which follows from the definitions Z|0〉=|0〉 and Z|1〉=−|1〉, with the total magnetization operator defined as

$$M=\sum_{j}Z_{j},$$

and the initial states constructed as described in Section 3.2.2. For the sector-*k* W state $|W_{N,k}\rangle$, we therefore have

$$\langle W_{N,k}\left|M\right|W_{N,k}|=\rangle N-2k.$$

We find perfect agreement between the benchmarked magnetization and the magnetization obtained by selecting the sector whose approximate ground-state energy is lowest. This procedure is directly aligned with the ideas of the KQD method, where one works within fixed-*k* (particle-number or magnetization) symmetry sectors and chooses an initial state residing in that sector before performing the Krylov-space diagonalization.

## Appendix C. Postprocessing: Magnetization Sector Filtering

During the field sweep hardware runs, we initially observed that several magnetization sectors reported identical (and correct) ground-state energy values, producing a flat behavior, unlike what is seen in Figure A1, while this is sufficient for estimating the ground-state energy, it prevents a reliable determination of the magnetization.

This effect arises from measurement errors: incorrect bitstrings do not alter the estimated minimum eigenvalue of the matrix elements, provided that all relevant correct bitstrings occur. However, such errors are detrimental for estimating the magnetization, which depends on the population of bitstrings rather than just the lowest energy.

Quantum hardware errors can occur anywhere in the circuit execution pipeline (gate imperfections, decoherence, readout noise, etc.). In the bitstring distribution shown in Figure A2, we observe that for a fixed-*k* evolution, where the model should conserve total excitation, some measured bitstrings nevertheless contain too few or too many excitations.

To address this, we discard all bitstrings whose excitation number does not match the initial one. Although this filtering step is wasteful, we found that it yields a reliable identification of the magnetization sector: only the physically correct sector exhibits the lowest post-selected energy.

## Appendix D. Setup and Parameterization of Calculations

### Appendix D.1. Quantum Simulations

All 1D quantum-hardware results in this work are run on ibm_marrakesh 156-qubits IBM Heron r2 superconducting qubits QPU. All 2D quantum-hardware results are run on ibm_miami, the 120-qubit IBM Quantum Nighthawk processor, which features a square lattice topology with four-nearest-neighbor connectivity designed to reduce SWAP-gate overhead in two-dimensional workloads.

Throughout all simulations we fix the time step to dt=0.06 and use the same initial state as described above. For the 1D Hamiltonians, to mitigate the non-sparsity of the ground state and reliably capture all relevant bitstrings, we employ 300,000 shots per Krylov dimension for the 18-qubit system and 600,000 shots for the 30-qubit system, with a total of 5 Krylov steps. These cost 7.5 and 15 min of quantum hardware time per sampling job accordingly, i.e., for each given value of the external field or Δ. For the 2D Hamiltonians, we use 100,000 shots per Krylov step, with a total of 7 steps, and 49 min of quantum hardware time per sampling job. At each step, the time-evolution operator is approximated by a second-order Trotter circuit with three repetitions. The results of the field sweep are summarized in Figure 4. To provide further insight into the sampling efficiency and hardware performance, Table A1 summarizes the bitstring statistics obtained from the 30-qubit ibm_marrakesh simulations. For a 30-qubit system, the full Hilbert space is exponentially large, spanning $2^{30}$ (approximately $1.07\times 10^{9}$) possible basis states. Despite this vast state space, SKQD successfully extracts the most physically important bitstrings required to construct the effective Krylov Hamiltonian in the large field regime. As outlined in Appendix B, we apply a post-selection filter to retain only the “filtered bitstrings,” which are those that strictly preserve the original Hamming weight (total excitation number) of the chosen initial state. The substantial difference between the total unique bitstrings sampled and the filtered bitstrings occurs because we did not apply any active hardware error mitigation techniques during these runs. Consequently, hardware noise induces population spillover into adjacent, non-physical magnetization sectors, as seen in Figure A2.

**Table A1:** Thirty sites ibm_marrakesh unique bitstrings used in the results from Figure 4. It is interesting to note that the sampling budget can be optimized as a function of the field, given that the number of shots required for convergence decreases because the ground state is supported by significantly fewer unique bitstrings.

Field Strength [T]	Unique Bitstrings	Filtered Bitstrings
0	2,614,501	346,579
1	2,594,156	328,272
2	2,621,787	413,509
3	2,614,781	436,112
4	2,607,803	432,877
5	2,624,556	448,407
6	2,636,541	433,806
7	2,628,267	426,417
8	2,696,189	419,326
9	2,630,196	312,631
10	2,581,484	260,838
11	2,587,489	245,419
12	2,536,459	188,716
13	2,426,888	128,911
14	2,213,621	22,302
15	2,173,180	3992

### Appendix D.2. DMRG Benchmark

To obtain essentially exact reference data for the ground-state energy and magnetization, we perform classical simulations using the density matrix renormalization group (DMRG) in a matrix–product–state (MPS) formulation. We consider a spin-$\frac{1}{2}$ chain of length *L* with open boundary conditions and couplings $J_{x}$, $J_{y}$, $J_{z}$ and a longitudinal field $h_{z}$, as implemented in the models of the TeNPy library [24]. Exploiting the underlying U(1) symmetry, we work in the $S^{z}$-conserving sector, and for each value of the longitudinal field we perform a sweep over total-magnetization sectors *q*, initializing the MPS from simple product states with the corresponding numbers of up and down spins. For every sector we run finite-system DMRG with a maximum bond dimension $\chi_{\max}=128$, up to four sweeps, and a truncation threshold for singular values of ${\mathrm{svd}}_{\min}=10^{-10}$. We then select the sector yielding the lowest variational energy as the ground state for that field value. From the converged MPS we extract both the ground-state energy and the corresponding total magnetization $\langle S_{\mathrm{tot}}^{z}\rangle$, which we use as classical benchmark data for the quantum algorithms studied in this work. To extend our benchmarks to 2D, we perform DMRG simulations on an 8×8 square lattice with open boundary conditions, modeling the quasi-2D Heisenberg anti-ferromagnet $\left[CuCl{\left(pyz\right)}_{2}\right]\left(BF_{4}\right)$ with an isotropic coupling J=0.81 meV [23]. The 2D geometry is mapped onto a 1D MPS snake-like path using the TeNPy library. To ensure convergence in this higher-dimensional setting, we employ a density matrix mixer and a multi-step bond dimension schedule, gradually increasing $\chi_{\max}$ from 10 to 400.

## References

[1] Yu H., Zhao Y., Wei T.C. (2023). Simulating large-size quantum spin chains on cloud-based superconducting quantum computers. Phys. Rev. Res..

[2] Keenan N., Robertson N.F., Murphy T., Zhuk S., Goold J. (2023). Evidence of Kardar-Parisi-Zhang scaling on a digital quantum simulator. npj Quantum Inf..

[3] Rosenberg E., Andersen T.I., Samajdar R., Petukhov A., Hoke J.C., Abanin D., Bengtsson A., Drozdov I.K., Erickson C., Klimov P.V. (2024). Dynamics of magnetization at infinite temperature in a Heisenberg spin chain. Science.

[4] Yoshioka N., Amico M., Kirby W., Jurcevic P., Dutt A., Fuller B., Garion S., Haas H., Hamamura I., Ivrii A. (2025). Krylov diagonalization of large many-body Hamiltonians on a quantum processor. Nat. Commun..

[5] Kumaran K., Sajjan M., Pokharel B., Wang K., Gibbs J., Cohn J., Jones B., Mostame S., Kais S., Banerjee A. (2025). Superdiffusion resilience in Heisenberg Chains with 2D interactions on a quantum processor. arXiv.

[6] Laurell P., Scheie A., Mukherjee C.J., Koza M.M., Enderle M., Tylczynski Z., Okamoto S., Coldea R., Tennant D.A., Alvarez G. (2021). Quantifying and Controlling Entanglement in the Quantum Magnet Cs_2_CoCl_4_. Phys. Rev. Lett..

[7] Hammar P.R., Stone M.B., Reich D.H., Broholm C., Gibson P.J., Turnbull M.M., Landee C.P., Oshikawa M. (1999). Characterization of a quasi-one-dimensional spin-1/2 magnet which is gapless and paramagnetic for *gμ*_B_*H* ≲ *J* and *k*_B_*T* ≪ *J*. Phys. Rev. B.

[8] Lake B., Tennant D.A., Caux J.S., Barthel T., Schollwöck U., Nagler S.E., Frost C.D. (2013). Multispinon Continua at Zero and Finite Temperature in a Near-Ideal Heisenberg Chain. Phys. Rev. Lett..

[9] Faure Q., Takayoshi S., Simonet V., Grenier B., Månsson M., White J.S., Tucker G.S., Rüegg C., Lejay P., Giamarchi T. (2019). Tomonaga-Luttinger Liquid Spin Dynamics in the Quasi-One-Dimensional Ising-Like Antiferromagnet BaCo_2_V_2_O_8_. Phys. Rev. Lett..

[10] Cui Y., Zou H., Xi N., He Z., Yang Y.X., Shu L., Zhang G.H., Hu Z., Chen T., Yu R. (2019). Quantum Criticality of the Ising-like Screw Chain Antiferromagnet SrCo_2_V_2_O_8_ in a Transverse Magnetic Field. Phys. Rev. Lett..

[11] Lehmann W.P., Breitling W., Weber R. (1981). Raman scattering study of spin dynamics in the quasi-1D Ising antiferromagnets CsCoCl_3_ and CsCoBr_3_. J. Phys. C Solid State Phys..

[12] Stoudenmire E., White S.R. (2012). Studying Two-Dimensional Systems with the Density Matrix Renormalization Group. Annu. Rev. Condens. Matter Phys..

[13] Haferkamp J., Hangleiter D., Eisert J., Gluza M. (2020). Contracting projected entangled pair states is average-case hard. Phys. Rev. Res..

[14] Yu J., Moreno J.R., Iosue J.T., Bertels L., Claudino D., Fuller B., Groszkowski P., Humble T.S., Jurcevic P., Kirby W. (2025). Quantum-Centric Algorithm for Sample-Based Krylov Diagonalization. arXiv.

[15] Alexeev Y., Amsler M., Barroca M.A., Bassini S., Battelle T., Camps D., Casanova D., Choi Y.J., Chong F.T., Chung C. (2024). Quantum-centric supercomputing for materials science: A perspective on challenges and future directions. Future Gener. Comput. Syst..

[16] Piccinelli S., Baiardi A., Barison S., Rossmannek M., Vazquez A.C., Tacchino F., Mensa S., Altamura E., Alavi A., Motta M. (2026). Quantum chemistry with provable convergence via randomized sample-based Krylov quantum diagonalization. arXiv.

[17] Rosanowski E.O., Eisinger J., Funcke L., Poschinger U., Schmidt-Kaler F. (2025). Sample-Based Krylov Quantum Diagonalization for the Schwinger Model on Trapped-Ion and Superconducting Quantum Processors. arXiv.

[18] Kanno K., Kohda M., Imai R., Koh S., Mitarai K., Mizukami W., Nakagawa Y.O. (2023). Quantum-Selected Configuration Interaction: Classical diagonalization of Hamiltonians in subspaces selected by quantum computers. arXiv.

[19] Robledo-Moreno J., Motta M., Haas H., Javadi-Abhari A., Jurcevic P., Kirby W., Martiel S., Sharma K., Sharma S., Shirakawa T. (2025). Chemistry beyond the scale of exact diagonalization on a quantum-centric supercomputer. Sci. Adv..

[20] Sugisaki K., Kanno S., Itoko T., Sakuma R., Yamamoto N. (2025). Hamiltonian simulation-based quantum-selected configuration interaction for large-scale electronic structure calculations with a quantum computer. Phys. Chem. Chem. Phys..

[21] Mikkelsen M., Nakagawa Y.O. (2025). Quantum-selected configuration interaction with time-evolved state. Phys. Rev. Res..

[22] Onah C., Firt R., Michielsen K. (2026). Empirical Quantum Advantage in Constrained Optimization from Encoded Unitary Designs. arXiv.

[23] Kubus M., Lanza A., Scatena R., Dos Santos L.H.R., Wehinger B., Casati N., Fiolka C., Keller L., Macchi P., Rüegg C. (2018). Quasi-2D Heisenberg Antiferromagnets [CuX(pyz)_2_](BF_4_) with X = Cl and Br. Inorg. Chem..

[24] Hauschild J., Unfried J., Anand S., Andrews B., Bintz M., Borla U., Divic S., Drescher M., Geiger J., Hefel M. (2024). Tensor network Python (TeNPy) version 1. SciPost Phys. Codebases.

